# Unexplained Inguinal Adenopathy Leading to the Diagnosis of Metastatic High-Grade Serous Ovarian Carcinoma

**DOI:** 10.7759/cureus.106024

**Published:** 2026-03-28

**Authors:** Olivia R Kaufman, Ishita Agarwal, Morgan W Stewart, Peighton E Davis, Brett Dunbar

**Affiliations:** 1 Medicine, Kansas City University of Medicine and Biosciences, Joplin, USA; 2 Clinical Anatomy, University of Iowa, Iowa City, USA; 3 General Surgery, Mercy Hospital Pittsburg, Pittsburg, USA

**Keywords:** atypical metastasis, extraperitoneal metastasis, high-grade serous ovarian carcinoma, inguinal mass, ovarian carcinoma metastasis

## Abstract

High-grade serous ovarian carcinoma (HGSOC) is the most common subtype of ovarian cancer, most commonly originating from the epithelium of the fallopian tube. In metastasis of HGSOC cells, there is a particular predilection for the omentum. Metastatic involvement of extraperitoneal lymph nodes, particularly the inguinal region, is rare and may pose a diagnostic challenge. We report a case of an 87-year-old female patient presenting with a right inguinal mass, in whom the pathology of the surgically removed mass confirmed the diagnosis of metastatic HGSOC, requiring oncological follow-up and management. This atypical pattern of spread underscores the importance of considering a Müllerian primary in the differential diagnosis of unexplained inguinal adenopathy and highlights the role of histopathology and immunohistochemistry in establishing the correct diagnosis.

## Introduction

Ovarian cancer continues to represent the most lethal gynecologic malignancy, with high-grade serous ovarian carcinoma (HGSOC) constituting the predominant and most aggressive histologic subtype, accounting for the majority of ovarian cancer-related deaths [1]. HGSOC is notable for its rapid progression and tendency to present at an advanced stage, as effective methods for early detection remain limited [2]. HGSOC is classified as a type II epithelial ovarian tumor, a category defined by aggressive clinical behavior and distinctive molecular alterations [3]. These tumors almost uniformly harbor TP53 mutations as well as germline mutations in BRCA1 and BRCA2, substantially elevating the risk of developing HGSOC [1,4]. The pattern of disease in HGSOC is typically characterized by dissemination within the peritoneal cavity, occurring through direct extension to adjacent structures or exfoliation of malignant cells from the primary tumor [1]. Although distant spread beyond the peritoneal cavity is uncommon, metastasis to pelvic and para-aortic lymph nodes may occur and represents an important pathway of disease progression. While lymph node involvement is generally associated with worse outcomes, isolated nodal disease has been linked to more favorable prognoses compared with extensive peritoneal metastasis or the presence of malignant ascites [5,6]. There are limited reports of atypical metastatic pathways of HGSOC, highlighting the extreme rarity of unusual metastatic locations and atypical presentations. Current screening approaches, including transvaginal ultrasonography combined with serum CA-125 measurement, have shown limited effectiveness and have not demonstrated a survival benefit [1,7]. As a result, maintaining a high index of clinical suspicion paired with genetic testing remains a critical strategy for identifying individuals with hereditary susceptibility, particularly those with a significant family history of breast or ovarian cancer.

## Case presentation

**Figure 1:**
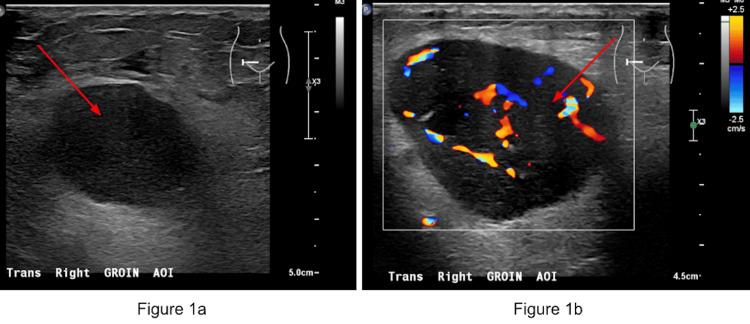
Pelvis limited non-obstetrical ultrasound of the right groin (red arrows) without Doppler (a) and with Doppler (b). The image demonstrates the right inguinal region with a vascularized, hypoechoic mass measuring up to 5.4 cm (red arrows).

An 87-year-old female with a history of benign hypertension, type 2 diabetes mellitus with stage 3a chronic kidney disease, mixed hyperlipidemia, and basal cell carcinoma of the right forearm, first presented to her primary care provider with the complaint of a groin mass in November 2025. She had been aware of this lump for approximately three to five days. The lump did not cause discomfort during physical activities such as walking, bending, or exercising, and was not associated with any pain or itching. She reported no recent urinary tract infections or yeast infections, with the last yeast infection occurring 30 years prior. She also reported no abdominal pain or diarrhea. There were no instances of fevers, chills, night sweats, rashes, itching, or unexplained weight loss. She had a history of water retention, treated with Lasix. Her colonoscopy results have always been normal. Physical exam findings showed a large mass in the right groin that was non-pulsatile, firm, and nontender. An ultrasound of the mass was ordered for further evaluation, demonstrating a 5.4 cm mass (Figure 1).

**Figure 2:**
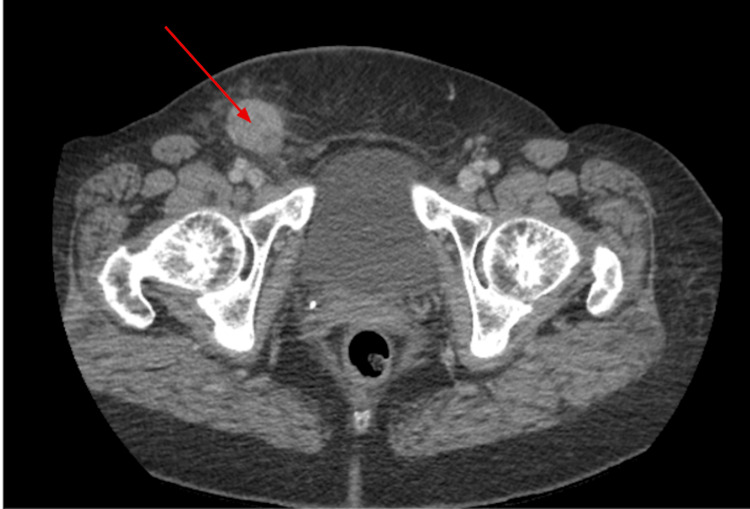
Axial computed tomography of the abdomen and pelvis with contrast demonstrating a right inguinal mass (red arrow). The image demonstrates a pathologically enlarged, enhancing right inguinal and right external iliac/obturator adenopathy, consistent with lymphoma or metastatic disease (red arrow).

The patient was then referred to a general surgery clinic for further evaluation about four days after her initial visit to her primary care provider. A CT of the abdomen and pelvis with contrast was ordered for further investigation, with findings of a right inguinal mass and adenopathy (Figure 2).

Approximately eight weeks later, general surgery performed an excision of the right groin mass with layered closure. The excised mass measured to be 5.5 x 4 x 4 cm. Pathology reported metastatic carcinoma consistent with metastatic HGSOC. Immunohistochemical evaluation of this patient's tumor for mismatch repair (MMR) proteins was performed to provide predictive information on the likelihood of response to immune checkpoint blockade therapy. MMR protein expression in this case was normal. Flow cytometry showed no evidence of a B or T cell lymphoproliferative disorder.

**Figure 3:**
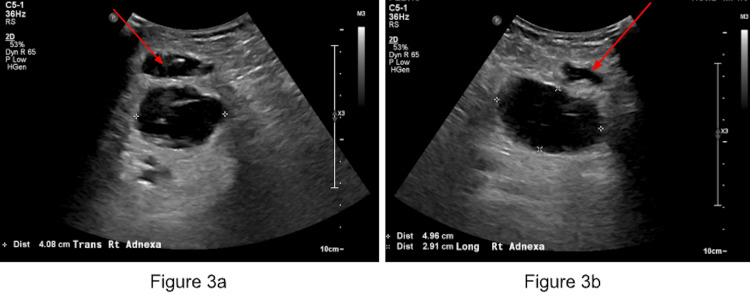
Transvaginal transverse (a) and longitudinal (b) ultrasound images of the right ovary demonstrating a mass (red arrows). This image demonstrates an enlarged right ovary with a complex hypoechoic mass in the right adnexa, measuring 5 cm (red arrows). This is superior to the incision site in the right groin, which could be a hematoma.

The patient was seen in the general surgery clinic the next week for follow-up. She had developed some discomfort and clear watery to yellow-tinged drainage post-procedurally that required changing several dressings, diagnosed as a seroma. She underwent subsequent testing, including a complete blood count (CBC), comprehensive metabolic panel (CMP), cancer antigen 125, PET scan, and transvaginal ultrasound. There were no pertinent findings on CBC or CMP, but cancer antigen 125 was found to be elevated (120 U/mL). A transvaginal ultrasound was conducted approximately two weeks postoperatively, demonstrating an enlarged right ovary with a hypoechoic mass (Figure 3).

**Figure 4:**
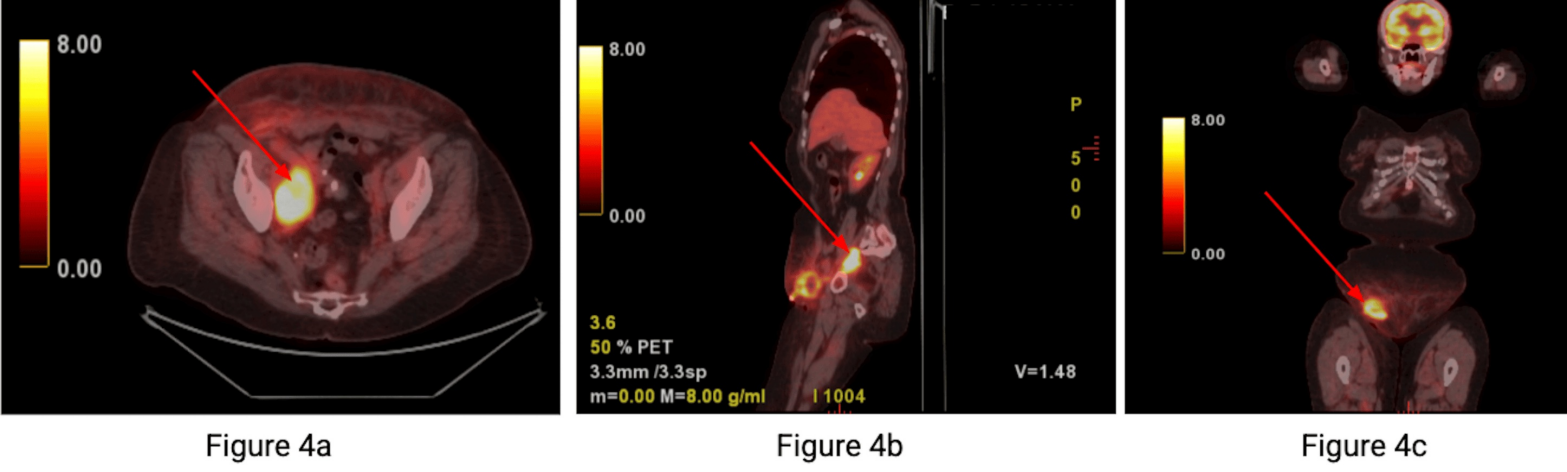
Positron emission tomography scan of the chest, abdomen, and pelvis with cross-sectional (a), sagittal (b), and coronal (c) views. These images demonstrate a 5.8 x 4.1 cm right pelvic sidewall/obturator mass (a and c) and a 2.7 x 1.8 cm retroperitoneal adenopathy (b), consistent with metastatic disease. Also note postoperative changes in the right inguinal region with some soft tissue edema.

Positron emission tomography (PET) scan demonstrated a hypermetabolic right pelvic mass measuring 5.8 x 4.1 cm with a maximum standardized uptake value (SUVmax) of 14.3, consistent with metastatic disease. Additional hypermetabolic right pericaval retroperitoneal lymphadenopathy was identified, measuring approximately 2.7 x 1.8 cm with an SUVmax of 8.0, also concerning for metastatic involvement. Surrounding soft tissue edema and fluid were visualized, with residual tumor not excluded. Mild symmetric metabolic activity was observed in the bilateral hilar regions, which is favored to represent reactive changes (Figure 4).

The patient then followed up with oncology within two weeks. Immunohistochemical testing was conducted (Table 1). The treatment plan determined by oncology is to initiate neoadjuvant chemotherapy, carboplatin/paclitaxel every 21 days for three cycles, follow-up imaging after three cycles to determine response to therapy, potential debulking surgery if chemotherapy response is favorable, and port placement. Approximately two weeks after the initial appointment with oncology, general surgery performed port placement. She continues to follow up with oncology for ongoing management.

**Table 1:** Immunohistochemical markers and results.

Marker	Result
CDX2	Negative
CK7	Strongly positive
CK20	Negative
Estrogen (ER)	Largely positive
GATA	Negative
p16	Patchy positive
p53	Diffusely and strongly positive
p63	Negative
Progesterone (PR)	Negative
Thyroglobulin	Negative
WT-1	Diffusely and strongly positive
Vimentin	Negative

## Discussion

This case describes the workup of an isolated finding of a nontender palpable inguinal mass discovered by the patient, which led to the diagnosis of HGSOC. This is an uncommon initial presentation, as HGSOC typically spreads via transcoelomic dissemination intraperitoneally, and extra-abdominal inguinal lymph node involvement is atypical [8,9].

This case of HGSOC was diagnosed by excision of the inguinal lymph node and subsequent pathology analysis. Initial imaging modalities failed to detect the primary tumor, which was identified later via PET/CT scan and transvaginal ultrasound. This sequence of diagnosis is uncommon and highlights the importance of immunohistochemical analysis of lymph node tissue.

A recent case series and literature review describes 51 reported cases of ovarian cancer with inguinal involvement, 40 of which presented with groin masses initially, and approximately 20 cases lacked the typical intraperitoneal spread of the primary tumor [5]. Thus, isolated superficial inguinal lymph node metastasis as the initial presenting symptom of HGSOC with minimal intraperitoneal involvement remains an uncommon phenomenon. Our case reinforces these observations, underscoring the need for high clinical suspicion and comprehensive diagnostic workup, including excisional biopsy and correlation with tumor markers and advanced imaging when encountering unexplained inguinal lymphadenopathy in females. The review discusses that isolated inguinal nodal metastasis may be associated with more favorable outcomes when compared to those with other distant metastases [5].

There are several proposed mechanisms of inguinal spread. The first proposed mechanism is that when the primary lymphatic drainage pathway is obstructed, tumor cells may undergo retrograde drainage to the pelvic region and superficial inguinal lymph nodes [5]. Another hypothesis states that there are three routes of lymphatic drainage from the ovaries in utero, of which one minor route consists of lymphatic vessels within the round ligament of the uterus that connect to the distal external iliac lymph nodes and superficial inguinal lymph nodes [10,11]. The authors hypothesized that this pathway may persist for a small percentage of females from embryogenesis to adulthood [10]. These explanations suggest that inguinal involvement may represent an alternative lymphatic route rather than a fundamentally different disease process. Inguinal lymph node involvement in HGSOC is characterized as extra-abdominal metastasis and staged as IVB disease using the FIGO (International Federation of Gynecology and Obstetrics) system [12]. However, patients with isolated inguinal node involvement may have outcomes similar to advanced nodal disease rather than typical stage IVB presentations [12]. This warrants further research into whether isolated inguinal node involvement carries the same prognosis. Many reported patients, including this patient, were only diagnosed post excision of the lymph node [5]. Reliance on initial imaging alone may delay identification of a malignancy if there is no obvious tumor identified. This case report reinforces that consideration of a primary Mullerian malignancy should be worked up for isolated inguinal lymphadenopathy in females, even with the absence of an obvious abnormality. This case adds to the literature describing HGSOC, which initially presented as isolated inguinal lymphadenopathy. The growing body of evidence around the recurring theme of inconclusive pelvic imaging and diagnosis made based on nodal biopsy may support the hypothesis that inguinal lymph node spread for primary ovarian tumors may be underrecognized.

Future research is needed to investigate routes of lymphatic spread for HGSOC and determine the implications of metastasis to superficial inguinal nodes. Larger-scale studies are needed to clarify tumor staging, prognosis, and clinical course of inguinal nodal metastasis versus intra-abdominal metastasis.

## Conclusions

This case highlights an unusual presentation of high-grade serous ovarian adenocarcinoma manifesting initially as an isolated inguinal mass. Because HGSOC most often spreads throughout the peritoneal cavity, extraperitoneal lymph node involvement can delay recognition and create diagnostic uncertainty. In this patient, excisional biopsy with immunohistochemical analysis was essential in establishing a diagnosis and guiding targeted imaging strategies, ultimately leading to identification of a primary ovarian mass.

This case reinforces several important clinical considerations. First, unexplained inguinal lymphadenopathy in females, especially when pathology reveals malignancy, should prompt consideration of a gynecologic primary lesion, including HGSOC. Second, tissue diagnosis remains imperative as imaging alone may not be able to identify the primary tumor early in the disease course. Third, awareness of atypical lymphatic pathways, such as potential spread via the round ligament, may help explain unusual metastatic patterns and prevent misclassification of disease origin. Although isolated inguinal metastasis from HGSOC remains rare, recognition of this pattern has relevant implications for staging, management, and prognostic assessment. Reporting of additional cases will be important to better characterize the biologic behavior and outcomes associated with this presentation. Ultimately, meaning a broad differential diagnosis and incorporating histopathology, tumor markers, and advanced imaging are critical to ensuring timely, accurate diagnosis and improving patient outcomes for those who present with atypical metastatic nodal disease.
